# K. A. Fall and S. Howard: Alternatives to domestic violence, a homework manual for battering intervention groups, 3rd revised edition

**DOI:** 10.1007/s00787-012-0272-y

**Published:** 2012-03-31

**Authors:** Nannet Buitelaar

**Affiliations:** de Waag Utrecht, Oudlaan 9, 3515 GA Utrecht, The Netherlands

This new version of *A homework manual for battering intervention groups* is a very convenient book. It was written for men who joined a battering intervention group or men who are concerned about some aspect of abuse in their lives. It contains exercises for all educational levels, to read and fill in at home and to be brought back to the group.

The book starts with describing myths and expectations about attending a therapy group, goal setting and defining abuse and battering. Then, the authors explain the continuum of controlling behaviours and violence and the difference between taking a time out and walking away. This is followed by some chapters about several aspects of relationships: trust, respect, accountability, sexuality, negotiating and communication. The book ends with some specific topics like parenting and the role of drugs and alcohol in domestic violence. The style of the book is clear and accessible. It contains a lot of information, stories of group members and exercises.

In the introduction, the authors mention that most of the professionals working in this field are trained in the Duluth model [1]. The Duluth model is based on the perception of intimate partner violence as a consequence of socially accepted opinions about using power and control by men in partner relationships. The purpose of the Duluth intervention is to change the opinions of the offender. It does not focus on psychiatric disorders or improving relationships.

The main criticism on the Duluth model is the lack of scientific evidence for this model and about creating a defensive attitude with the participants focusing on acknowledging the incorrectness of their opinions. The method is not very motivating to change behaviour.

The authors adapt the Duluth model without criticising it. However, they say that they added some different techniques to the former model to make some creative expansions. They integrated feedback from a lot of group members and group leaders to make it more suitable to daily life. A lot of patient stories are told, so that men can identify easily. The reader is addressed in a friendly way and men are encouraged to change. Moreover, they added exercises (with a tool icon) for learning new skills as they recognise that lacking social and relational skills is a major problem for most of the men in these groups. Especially the “cooperating through good communication” is about learning new skills. It contains explanations and exercises about rules for communication, reflective listening and assertiveness. These are very important skills for maintaining a good relationship.

Although they do not explicitly say so, it feels like what the authors call “different techniques” are in fact some elements of other therapeutic models for treating intimate partner violence such as individualized cognitive behaviour therapy (Individualized CBT) [2], motivational interviewing [3] and solution-focused treatment [4].

These models share a focus on strengths of people, solutions instead of problems and building skills. Further, they focus on enhancing motivation by using specific interventions like expressing empathy, avoiding argumentation, using key questions, reflective listening and eliciting language that promotes change. In addition, Individualized CBT has a focus on individual psychopathology.

The authors did a very good job by inserting some of the elements of these other therapeutic models, but they could have done it more consequently. My main concern with this book is that it is rather problem oriented and focuses on insight and acknowledgement of the incorrectness of the behaviour and opinions of the clients.

For example, the formulation of the chapter titles in a negative way, like “defining abuse and battering” and “exploring and defeating intimidation” is problem oriented. Fortunately, they did well with chapter titles like: “giving and receiving respect” and “creating a trusting relationship”.

Another example of focusing on problems instead of solutions is the fact that clients are never, in this book, asked about their capacities and what they are doing well in their relationships. Exploring strength and capacities should at least be added to the chapter of goal setting, for example. A typical solution-focused way of asking would be: can you describe a situation when you did not use violence? What does it say about you that you managed that time to NOT use violence? What can you do to act more often like this? Actually all chapters which want to achieve changing behaviour can also use some of these questions.

Although the authors added exercises for learning new tools, they could have done this more often. The chapter Maintaining Positive Sexual Relationships focuses strongly on building empathy for victims of rape or sexual assault. It addresses too little the problems men can have with their own sexuality and intimacy in their relationship and how to solve or discuss these problems with their partners. As mentioned before, the chapter “cooperating through good communication” is a very good example of solution-focused working and will help these clients a lot.
